# Inorganic Arsenic and Human Prostate Cancer

**DOI:** 10.1289/ehp.10423

**Published:** 2007-11-08

**Authors:** Lamia Benbrahim-Tallaa, Michael P. Waalkes

**Affiliations:** Inorganic Carcinogenesis Section, Laboratory of Comparative Carcinogenesis, National Cancer Institute at the National Institute of Environmental Health Sciences, National Institutes of Health, Department of Health and Human Services, Research Triangle Park, North Carolina, USA

**Keywords:** androgen-independent, AR, arsenic, carcinogenesis, DNA methylation, human malignant transformation, MAP kinase, prostate, Ras

## Abstract

**Objective:**

We critically evaluated the etiologic role of inorganic arsenic in human prostate cancer.

**Data sources:**

We assessed data from relevant epidemiologic studies concerning environmental inorganic arsenic exposure. Whole animal studies were evaluated as were *in vitro* model systems of inorganic arsenic carcinogenesis in the prostate.

**Data synthesis:**

Multiple studies in humans reveal an association between environmental inorganic arsenic exposure and prostate cancer mortality or incidence. Many of these human studies provide clear evidence of a dose–response relationship. Relevant whole animal models showing a relationship between inorganic arsenic and prostate cancer are not available. However, cellular model systems indicate arsenic can induce malignant transformation of human prostate epithelial cells *in vitro*. Arsenic also appears to impact prostate cancer cell progression by precipitating events leading to androgen independence *in vitro*.

**Conclusion:**

Available evidence in human populations and human cells *in vitro* indicates that the prostate is a target for inorganic arsenic carcinogenesis. A role for this common environmental contaminant in human prostate cancer initiation and/or progression would be very important.

Inorganic arsenic, a metalloid, is ubiquitously distributed in nature. In natural deposits, this metalloid forms a complex with pyrite, for which it has a strong affinity (Nordstrom 2002). However, under certain conditions (pH, temperature, etc.), inorganic arsenic readily dissociates from its soil-bound forms and enters the aquifer (Smedley and Kinniburgh 2002). For this reason, the major source of human exposure to arsenic is naturally contaminated drinking water from underground wells. Probably more than 100 million people are exposed to inorganic arsenic at levels above 10 μg/L, the drinking-water standard in many countries [International Agency for Research on Cancer (IARC) 2004]. Arsenic is also released into the atmosphere from both natural and anthropogenic sources. Globally, natural emissions of arsenical compounds have been estimated at about 8,000 tons each year, whereas anthropogenic emissions are about 3 times higher [National Research Council (NRC) 1999, 2001]. Food, particularly vegetables and rice, may be an additional source of exposure to inorganic arsenic (NRC 1999, 2001). Occupational exposure to arsenic occurs in specific industries such as mining, smelting operations, wood preservation, and electronics (IARC 2004).

Arsenical exposure produces various adverse effects such as dermal lesions, hypertension, ischemic heart disease, liver disease, peripheral vascular disorders, arteriosclerosis, diabetes, neuropathy, and cancer (NRC 1999, 2001). The carcinogenic potential of inorganic arsenic exposure through drinking water in humans is a cause for considerable concern [IARC 2004; NRC 1999, 2001; National Toxicology Program (NTP) 2004]. Indeed, inorganic arsenic is a potent, multisite human carcinogen most frequently associated with tumors of the skin, urinary bladder, and lung (IARC 2004; NRC 1999, 2001; NTP 2004). There are also human data associating inorganic arsenic exposure with cancers of the liver, prostate, and kidney. The mechanisms by which inorganic arsenic is carcinogenic are not completely defined (Kitchin 2001; Rossman 2003; Simeonova and Luster 2000; Waalkes et al. 2007). A challenge to elucidating these mechanisms has been the difficulty encountered in the development of experimental whole animal models of arsenic carcinogenesis. In essence, it has proven difficult until recently to induce cancer in animals using inorganic arsenic as a single agent (Waalkes et al. 2007). In place of whole animal models, cell lines such as human prostate epithelial cells (Achanzar et al. 2002), keratinocytes (Pi et al. 2003), and urothelial cells (Sens et al. 2004), which may represent *in vivo* targets of arsenic, provide a relevant and reasonable *in vitro* approach to study the molecular events in inorganic arsenic carcinogenesis.

## Arsenic toxicokinetics and metabolism

The metabolism of arsenic compounds in mammals has been reviewed (Aposhian and Aposhian 2006; Styblo et al. 2002; Thomas et al. 2001). Inorganic arsenic is well absorbed from the gastrointestinal tract and distributed throughout the body (NRC 2001). It freely crosses the rodent and human placenta (NRC 1999). In many tissues inorganic arsenic is biotransformed by methylation (NRC 2001). Some cells methylate inorganic arsenic very poorly or not at all, for example, keratinocytes (Patterson et al. 2003) or prostate epithelial cells (Benbrahim-Tallaa et al. 2005a). Biomethylation of arsenic is no longer considered a detoxification process, as trivalent methylated arsenical intermediates are highly toxic (Mass et al. 2001; Styblo et al. 2000; Wei et al. 2002) and possibly carcinogenic (Bredfeldt et al. 2006). Reduction of arsenate (As^5+^) to arsenite (As^3+^) is necessary before methylation can occur. Arsenate is rapidly reduced to arsenite by glutathione *S*-transferase omega and/or arsenate reductase. Arsenite is then methylated to form methylarsonate (MMA^5+^) and dimethylarsinic acid (DMA^5+^) by arsenic methyltransferase using *S*-adenosylmethionine (SAM) as the methyl donor. The intermediate metabolites methylarsonous acid (MMA^3+^) and dimethylarsinous acid (DMA^3+^) are generated during this process (Aposhian and Aposhian 2006; Styblo et al. 2000). The precise role of trivalent methylated arsenical species in inorganic arsenic carcinogenesis is not fully understood, although MMA^3+^ can induce malignant transformation of human urothelial cells *in vitro* (Bredfeldt et al. 2006).

Both arsenite and arsenate are actively transported into cells (Huang and Lee 1996; Liu et al. 2002) by mechanisms that may involve organic ion transporters (Bridges and Zalups 2005). Recent evidence indicates that multidrug resistance protein 1 (MRP1), an ATP-binding cassette transport protein, is involved in efflux of arsenite in an ATP- and glutathione-dependent manner. It appears arsenic is effluxed as a triglutathione complex (Leslie et al. 2004) produced by glutathione *S*-transferase pi (Liu et al. 2001), which may stress cellular redox systems from continuous demands on glutathione.

## Prostate cancer

The prostate gland is characterized by the age-dependent development of abnormal proliferative diseases ranging from benign prostate hyperplasia to overt malignancies. Prostate cancer is the most frequently diagnosed non-skin cancer among men and the second leading cause of male cancer deaths in the United States (Crawford 2003). There were approximately 6.7 million cancer deaths worldwide in 2002, and of these, prostate cancer was the fifth most common overall and the second most common among men (Parkin et al. 2005). Migrant studies provide strong evidence for the role of the environment in prostate cancer. Throughout the last several centuries, major migratory movements of humans have taken place in many parts of the world and continue even today. With increasing length of stay, cancer mortality rates among immigrants move toward those in the adopted country. This has been clearly shown for prostate cancer (McKay et al. 2003). There are also intra- and interracial differences in prostate cancer incidence and mortality rates worldwide, and the environment and migration patterns seem to influence these disparities (Moradi et al. 1998; Stemmermann et al. 1991; Thomas and Karagas 1996). These studies provide insight into the relative contributions of heredity and environment in prostate cancer.

## Inorganic arsenic as a human carcinogen

The NTP and the IARC have concluded that arsenic is a human carcinogen (IARC 2004; NTP 2004). Arsenic contamination of drinking water is a common occurrence and a worldwide public health issue. Some countries have truly daunting issues with arsenic contamination of drinking-water supplies, and endemic chronic arsenicalism is observed in many places in India, Bangladesh, Taiwan, and China (IARC 2004). Although chronic arsenic exposure produces a variety of adverse effects, its carcinogenic potential in humans is perhaps of greatest concern. Although the exact modes of action remain to be defined, it is reasonable to assume that site-specific and multifactorial mechanisms apply to inorganic arsenic.

The carcinogenic potential of arsenic was recognized over 100 years ago by Hutchinson (1887), a British physician who observed skin cancers occurring in patients treated with medicinal arsenicals. Further evidence for arsenic as a human carcinogen after industrial exposure comes from studies of arsenic ore smelters and pesticide workers (Brown and Ross 2002). In numerous countries it has been shown that people who consume arsenic-contaminated drinking water can develop various cancers (IARC 2004; NRC 1999, 2001; NTP 2004). Thus, arsenic is a human carcinogen after environmental, occupational, or medicinal exposures. Strong epidemiological associations exist between inorganic arsenic ingestion and cancers of skin, urinary bladder, and lung. Epidemiologic evidence has also linked arsenic in the drinking water to prostate, kidney, and liver cancers (IARC 2004; NRC 1999, 2001). In fact, in its 2004 evaluation summary, the most recent IARC monograph on arsenic clearly states “Excess mortality from prostate cancer was found in South-West Taiwan” (IARC 2004).

Data on concentrations of arsenic in human target tissues, especially for internal organs, are largely lacking. This factor becomes problematic when attempting to produce biokinetic models or when defining what are reasonable exposures for *in vitro* studies. At least some human tissues, particularly the skin, clearly will accumulate arsenic, and skin levels in the range of 5,700 μg/kg (∼76 μM) have been reported from arsenic intoxicated people in Bangladesh (IARC 2004). This is in contrast to circulating levels of up to 60 μg/L (∼ 0.8 μM) in blood and 274 μg/L (∼ 3.6 μM) in urine during chronic arsenic intoxication (NRC 1999). Thus, it is unclear if circulating or excreted levels of arsenic actually reflect target tissue or target cell burden. Perhaps most important, there is essentially no information on arsenic levels in the human prostatic tissue. Clearly, further work in this area is required.

## Arsenic carcinogenesis in animals

Until recently, inorganic arsenic in rodents was generally not carcinogenic except in model systems involving co-administration with known carcinogenic agents (Germolec et al. 1998; Rossman et al. 2004). However, a series of studies from our laboratory [for review, see Waalkes et al. (2007)] has recently demonstrated that inorganic arsenite administered during the second half of gestation to pregnant mice of several strains will induce or impact the development of cancer in the offspring as adults in various tissues, including tissues that are potential human targets such as liver and lung. In studies using prenatal arsenic exposure combined with exposure to additional agents after birth, tumors of the urinary bladder can also be induced. Together these studies provide consistent evidence that *in utero* arsenic is carcinogenic in mice and targets several tissues that are concordant with human target sites.

However, prostate cancers do not develop in these mouse studies (Waalkes et al. 2007). In this regard, the genetically unaltered mouse is not the rodent of choice for *in vivo* models of human prostate cancer (Shirai et al. 2000). The reasons for this include the observation that mice are resistant to the induction of prostatic tumors by chemical carcinogens as well as differences in anatomy and pathophysiology (Shirai et al. 2000). Transgenic mouse lines are available in which prostate carcinomas preferentially occur (Green et al. 1998; Shirai et al. 2000), but arsenic has not been tested in these models. Rats generally are considered a better rodent model of prostate cancer because prostate lesions can be chemically induced and in the early stages are androgen dependent (Shirai et al. 2000). However, arsenic bio-kinetics in rats is very dissimilar to that in humans or mice, and rats are considered a poor model for human arsenic toxicology (Aposhian and Aposhian 2006). Furthermore, although pentavalent methylated arsenicals are complete carcinogens and tumor promoters in rats (Wanibuchi et al. 2004), they do not target the prostate. Thus, at present, whole rodent prostate models of inorganic arsenic carcinogenesis are not available.

## Arsenic Exposure and Human Prostate Cancer

The first evidence that inorganic arsenic was associated with prostate cancer in humans came from Taiwan in the late 1980s (Chen et al. 1988; Table 1). This was a follow-up study that focused on dose–response relationships between arsenic and cancer in a population exposed to high levels of arsenic in the drinking water from local artesian wells. The population studied was from the area of endemic “blackfoot” disease in southwest Taiwan, a disease involving the peripheral vascular dysfunction likely due, at least in part, to arsenic exposure (Chen et al. 1985). Although the original study had not looked at cancer of the prostate (Chen et al. 1985), the subsequent study found a remarkable association between arsenic exposure and prostate cancer mortality in this population (Chen et al. 1988). In this regard, the age-standardized mortality from prostate cancer in the group exposed to the highest levels of arsenic in the drinking water (≥0.60 ppm) was nearly 6-fold greater than that of the general population in Taiwan. In addition, when drinking-water arsenic levels were stratified (< 0.30 ppm, 0.30–0.59 ppm and ≥0.60 ppm), a significant dose–response relationship occurred between arsenic level and age-adjusted prostate cancer mortality. The exposed population lived in a relatively small area and had similar lifestyles, diets, living conditions, and sociodemographic characteristics compared with those of nearby unaffected villages, prompting the authors to conclude that the striking differences in cancer mortality between these groups could be explained “solely by the difference in arsenic concentrations in drinking water” (Chen et al. 1988).

Prostate cancer is not always fatal, particularly in its early stages, and as the cause of death was determined in this study by death certificate (Chen et al. 1988), it is likely that the rate of deaths would be much lower than the incidence of prostate cancers in this population. There were also large increases in mortality from liver, lung, skin, bladder, and kidney cancers in this population due to arsenic exposure that generally exceeded the rate of prostate cancer deaths (Chen et al. 1988). Therefore, other cancers may have overshadowed relatively rare cancers of the prostate. Furthermore, prostate cancer is usually a disease of older men, and because arsenic is a very effective, multisite carcinogen, perhaps some of the most sensitive subjects may have died of other arsenic-induced cancers before the development of advanced and deadly prostate cancer. Indeed, prostate cancer is considered to have a relatively low case-fatality rate (IARC 2004), making mortality as an end point potentially insensitive of actual disease status, at least in the early stages.

A follow-up study to those of Chen et al. (1985, 1988) concerning arsenic and cancer mortality used some of the same population at risk but added data from additional villages in the area of endemic blackfoot disease and specifically studied dose–response relationships (Wu et al. 1989). In this study, the age-adjusted mortality for prostate cancer in the population exposed to the highest arsenic levels in the drinking water (≥0.60 ppm) was nearly 10-fold higher (9.18 deaths/100,000) than that at the lowest level (< 0.30 ppm; 0.95 deaths/100,000) of exposure. A clear dose–response relationship also occurred between arsenic exposure and prostate cancer mortality when drinking-water levels of arsenic were stratified (< 0.30, 0.30–0.59, and ≥0.60 ppm) in this study. These interpretations must be tempered by the small number of cancer deaths due to prostate cancer in this study, but, nonetheless, the findings are consistent with the prior work (Chen et al. 1988). Exposure levels were determined by median village levels of arsenic in drinking-water wells, and, as such, may be subject to the “ecological fallacy” that the association observed at the village level may not hold at the individual level (Wu et al. 1989). Even after considering this and other confounding factors, the authors felt that arsenic content should still be strongly suspected as the main cause of excess cancer deaths in this population (Wu et al. 1989).

In subsequent work from Taiwan, the study population was expanded from the area of endemic blackfoot disease used in the first two studies (Chen et al. 1988; Wu et al. 1989) to a much more comprehensive study of all 314 precincts and townships in Taiwan as a whole. In all, 83,656 wells were tested for arsenic (Chen and Wang 1990). Based on multiple regression analysis with adjustments for urbanization and age, mortality rates from cancer of the prostate again increased in correlation with increasing average drinking-water level of arsenic.

In an independent study of the area of endemic blackfoot disease in southwest Taiwan, Tsai et al. (1999) computed age-adjusted standardized mortality ratios (SMRs) using death certificates with national reference rates. The SMR for prostate cancer in the arsenic-exposed population was 1.96, with a 95% confidence interval (CI) of 1.4–2.6, indicating a significant increase in the number of observed cases compared with the number of expected based on the national reference rates. The number of observed cases in this arsenic-exposed population was 48, and dose–response effects were not investigated.

The role of drinking-water arsenic in prostate cancer mortality has also been studied in a U.S. population (Lewis et al. 1999). Mortality was assessed in a retrospective cohort of Millard County, Utah, residents along with drinking-water arsenic exposure levels that accounted for residence time in the study area. The cohort consisted of 2,073 members with at least 20 years of exposure history and was assembled through membership records of the Church of Jesus Christ of Latter-day Saints. Arsenic exposure was stratified into low (< 1,000 ppb-years), medium (1,000–4,999 ppb-years) and high (≥5,000 ppb-years) levels (Lewis et al. 1999). Without considering specific arsenic exposure levels, the overall SMR for prostate cancer mortality was significantly elevated in the cohort (1.45; 95% CI, 1.07–1.91, based on 50 deaths) compared with that of Utah white males. The authors indicate that SMR analysis hinted at a dose–response relationship when based on low (SMR = 1.07), medium [1.70 (significantly elevated)] and high (1.65) arsenic exposure (Lewis et al. 1999).

In a study from Australia, geographic areas with soil arsenic > 100 mg/kg and/or drinking-water concentrations > 0.01 mg/L were selected and related to cancer incidence (Hinwood et al. 1999). Standardized incidence ratios (SIRs) were generated for 22 areas of elevated arsenic exposure in Victoria and compared with all Victorian cancer rates as a baseline. For all areas with any elevated arsenic (soil or water or both), the SIR was significantly increased for prostate cancer (1.14; 95% CI, 1.05–1.23). Exposure was also stratified as only high soil or only high water arsenic (low) or both high soil and high water arsenic (high). When arsenic exposure was stratified by exposure type (i.e., high water only, high soil only, high water/high soil), the SIR for prostate cancer remained significantly elevated (1.20; 95% CI, 1.06–1.36), in the high water/high soil category. Dose–response analysis was performed on data stratified based on water content of arsenic as low (< 0.01 mg/L), medium (0.01–0.1 mg/L), high (0.1–0.2 mg/L), and very high (> 0.2 mg/L) levels. No linear dose response was detected for prostate cancer incidence using this water stratification, but based on graphical presentation, the SIRs for the high and very high categories appeared elevated (95% CIs did not include 1.0). The study included 619 cases of prostate cancer. The authors make the point that of those targets expected *a priori* from other studies, only prostate cancer was significantly elevated.

In a population of male copper foundry workers industrially exposed to arsenic as well as other metals, a correlative survey of plasma neoplastic biomarkers was conducted (Szymanska-Chabowska et al. 2004). A strong positive correlation occurred between urinary arsenic concentration and serum prostate-specific antigen (PSA). PSA is a well-established biomarker for prostate cancer that is considered a mainstay of early prostate cancer detection. The exposure to other metals complicates interpretation of this study, but the correlation between arsenic in the urine and circulating PSA was robust. In this regard, tumors arising from human prostate epithelial cells transformed by inorganic arsenic *in vitro* also show a remarkable overexpression of PSA (Achanzar et al. 2002).

The results of various positive studies of prostate cancer and arsenic exposure were considered as a whole by the IARC (2004). The specific conclusion was that “data from southwest Taiwan indicate a consistent pattern of increased mortality from prostate cancer in areas with high contamination by arsenic, and there is evidence of a dose-related effect” (IARC 2004). Although the prostate was not specifically mentioned as a human target site in the final evaluation of the monograph, the implications of the text are clear and, at least in part, are supported by the data from the United States and Australia, which make it less likely that the Taiwanese are uniquely sensitive. Whatever the conclusion, the available evidence indicates an obvious need for additional studies of arsenic as a human prostatic carcinogen.

As a potential complicating factor in dose–response analysis, evidence indicates that arsenic can adversely affect testicular function in animals, even at levels near the range for some human exposure situations. This includes loss of testicular weight, diminished sperm count, and decreased 17β-hydroxysteroid dehydrogenase (17β-HSD) activity in mice chronically given 4 ppm arsenic in the drinking-water (Pant et al. 2004). In this regard, 17β-HSD is an enzyme important in production of testosterone from its immediate precursors, such as androstenedione. Similarly, in rats chronic oral arsenic exposure decreases testicular weight, sperm count, testicular 17β-HSD activity, and plasma and testicular testosterone concentrations (Jana et al. 2006). Prostate cancer, particularly in its early stages, is dependent typically on circulating androgens and will regress with orchiectomy and/or antiandrogen therapy, two strategies commonly used in prostate cancer treatment (Kyprianou and Isaacs 1988). Thus, if higher doses of arsenic similarly suppressed testosterone production in humans, this could complicate the dose–response analysis by potentially diminishing carcinogenic response at higher doses. There is no direct evidence of this in humans, however.

## *In Vitro* Model of Arsenic-Induced Prostatic Carcinogenesis

*In vitro* models can be invaluable for studies on carcinogenic mechanisms and can be applied to the various stages of oncogenesis including initiation and progression. In fact, those employing human cells can provide carefully controlled exposure circumstances that are impossible in environmentally exposed human populations. Cell model systems have been used to identify molecular markers of transformation during prostate cancer development. In this regard, the majority of commonly used human prostate cell lines are derived from biopsies of metastatic prostate cancer (Webber et al. 1996a, 1997) and, as such, would be more appropriate for defining molecular events occurring during tumor progression to advanced prostate cancer. Prostate cancer has the added aspect of acquired androgen independence, generally occurring as a progression to a deadly form of the disease. Hence, tumor-derived cell lines have been used to extensively study androgen independence (Gustavsson et al. 2005). For the study of carcinogenic initiation, one would want a nontransformed (“normal”) line that is nontumorigenic upon inoculation into mice. Human arsenic exposure is typically to an acutely tolerable dose over long periods of time. To use doses (concentrations) similar to human exposure, cells should be exposed to relatively low levels of arsenic for protracted periods. Hence, an immortalized cell line is essential.

The human prostate epithelial cell line RWPE-1 was originally derived from normal human prostate epithelium (Bello et al. 1997; Webber et al. 1997). RWPE-1 cells are immortalized and nontumorigenic upon inoculation into immunocompromised mice, an important observation, as the ability to form tumors is a key element in the definition of cellular malignant transformation. By continuous exposure of this line to low levels of inorganic arsenic over a period of several months, a malignant transformant was developed (Achanzar et al. 2002). Essentially, RWPE-1 cells were cultured in the presence of 5 μM arsenic continuously for up to 30 weeks, while parallel control cultures served as passage-matched controls. Cell samples were frozen periodically to allow for assessment of time-course changes after confirmation of transformation. This chronic arsenic-exposed prostate epithelial (CAsE-PE) cell line, showed a 2.2-fold increase in matrix metalloproteinase-9 (MMP-9) secretion compared with control (Achanzar et al. 2002). Increased MMP-9 is associated with Ras-induced or cadmium-induced malignant transformation of RWPE-1 cells (Achanzar et al. 2001; Webber et al. 1996b), occurs in human prostate tumors and in primary cultures of prostatic cancer cells, and is associated with aggressive prostatic malignancies (Hamdy et al. 1994). When CAsE-PE cells were inoculated into the renal capsule of nude mice, all of the mice inoculated developed tumors within 10 weeks while control cells remained nontumorigenic (Achanzar et al. 2002). The aggressive carcinoma that developed from CAsE-PE inoculation showed several characteristics in common with human prostatic cancers, including overproduction of human PSA (Achanzar et al. 2002), clearly indicating their origin. The rapidly formed tumors resulting from CAsE-PE cell inoculation often invaded local tissue (Achanzar et al. 2002). Because animal models for arsenic carcinogenesis are currently absent for the prostate, this *in vitro* system has been used to help define the molecular events in arsenic-induced prostatic carcinogenesis. Indeed, these cells and their heterotransplantation tumors show a remarkable series of characteristics in common with human prostate carcinoma (Table 2). The finding that human prostate epithelial cells are directly susceptible to arsenic-induced malignant transformation strongly fortifies the evidence for a potential role of arsenic in human prostate cancer.

## Molecular Events in Arsenic-Induced Malignant Transformation

Several studies were conducted to examine the molecular events in arsenic-induced malignant transformation in human prostate cells, including studies on DNA methylation (Benbrahim-Tallaa et al. 2005a). Inorganic arsenic biomethylation uses SAM as the methyl donor, and SAM depletion can induce DNA hypomethylation (Loenen 2006). Indeed, in CAsE-PE cells arsenic-induced malignant transformation also induces genomic DNA hypomethylation (Benbrahim-Tallaa et al. 2005a). A decrease of DNA methyltransferase activity is an early event occurring before malignant transformation and may account for the subsequent genomic DNA hypomethylation (Benbrahim-Tallaa et al. 2005a). Arsenic-induced DNA hypomethylation occurs in malignantly transformed rodent liver cells (Zhao et al. 1997) and in the liver of mice after chronic exposure to inorganic arsenic (Chen et al. 2004). Furthermore, hepatocellular carcinoma induced by transplacental exposure to inorganic arsenic in mice is associated with aberrant gene expression changes likely due, at least in part, to errors in DNA methylation including hypomethylation of steroid signaling transcription factors (Waalkes et al. 2004). The finding of arsenic-induced DNA hypomethylation in human prostate cells indicates this may be a plausible contributing factor for tumor development in arsenic-exposed human populations (Benbrahim-Tallaa et al. 2005a). Carcinogenesis can result from aberrations of genomic DNA methylation that include hypomethylation of the promoter of cancer-related genes. Global hypomethylation of genomic DNA is often observed in tumors and contributes to overexpression of proto-oncogenes, growth factors, and genes that are involved in cancer cell proliferation, invasion, and metastasis (Stirzaker et al. 2004). DNA hypomethylation is viewed as a nongenotoxic mechanism facilitating aberrant gene expression (Counts and Goodman 1995; Vorce and Goodman 1989). Aberrant gene expression is a common occurrence in arsenic-exposed cells.

Studies show that both CAsE-PE and parental cells have a very poor capacity to methylate arsenic, making competition for SAM an unlikely basis for arsenic-induced DNA hypomethylation (Benbrahim-Tallaa et al. 2005a). There is, however, emerging evidence that during cellular adaptation to chronic arsenic exposure, SAM recycling may be reduced in order to overproduce glutathione for arsenic efflux through transsulfuration of homocysteine (Coppin et al. 2007).

A marked overexpression of unmutated K*-ras* was also observed in CAsE-PE cells (Benbrahim-Tallaa et al. 2005a). Although hypomethylation of the *ras* gene can lead to activation, the K*-ras* promoter region, including the major transcriptional initiation site, was essentially unmethylated in both control and CAsE-PE cells (Benbrahim-Tallaa et al. 2005a). Thus, although genomic DNA hypomethylation was observed in arsenic-transformed cells, this does not appear to be the direct cause of overexpression of K*-ras* (Benbrahim-Tallaa et al. 2005a). Whatever the basis, K*-ras* overexpression appears to have been a key molecular change associated with arsenic-induced transformation of CAsE-PE cells. K*-ras* overexpression was observed as early as 12 weeks after arsenic exposure and reached its highest level after approximately 30 weeks of continuous arsenic exposure, the time point for malignant transformation. This is consistent with previous data suggesting that K*-ras* amplification could be an early event in the pathogenesis of prostatic carcinogenesis (Lau et al. 2004) and may be a critical factor that drives prostate cancer development (Weber and Gioeli 2004). Thus, the *in vitro* prostate model of arsenic carcinogenesis (Benbrahim-Tallaa et al. 2005a) duplicates this key aspect of the corollary disease in humans (Table 2).

The normal development, growth, and survival of the prostate epithelium are regulated both by systemic and local androgen and by local production of growth factors by the prostatic stroma (Feldman and Feldman 2001). However, regulatory interactions between androgens and growth factors often become distorted in prostate cancer (Feldman and Feldman 2001). Ras is a critical signaling molecule that controls several signaling pathways in prostate cancer (Gioeli 2005; Weber and Gioeli 2004). Yet, *ras* mutations are infrequent in prostate cancer (Carter et al. 1990). This is consistent with the hypothesis that wild-type *ras* is chronically activated by autocrine and paracrine factor stimulation in prostate cancer (Gioeli 2005; Weber and Gioeli 2004). Virtually all the growth factor receptors upregulated in prostate cancer activate *ras* for their signal transduction activity (Gioeli 2005). In essence, *ras* signaling represents a convergence point for numerous diverse extracellular signals in prostate cancer (Gioeli 2005). One of the best-characterized effector pathways triggered by Ras activation is the MAPK (serine–threonine protein kinases) pathway. The activation of K*-ras* by arsenic in CAsE-PE cells is by some mechanism other than promoter region hypomethylation, perhaps involving genes upstream of *ras* (Benbrahim-Tallaa et al. 2005a). In this regard, a series of proteins participating in protein–protein interactions are responsible for the control of *ras* activation and include Raf (c-Raf-1, A-Raf, and B-Raf), MEK (MAPK/ERK kinases 1 and 2), and ERK1/2 (McCubrey et al. 2006). The ERK1/2 signaling pathway plays an important role in cellular growth and differentiation (McCubrey et al. 2006). Thus, molecular events upstream of *ras* have been compared in CAsE-PE and control cells. Clearly, proteins upstream of K*-ras*, including A-Raf and B-Raf showed greatly increased expression in CAsE-PE cells compared with control (Benbrahim-Tallaa et al. 2007). There was also an increased expression of phosphorylated MEK1/2 and ELK in CAsE-PE cells compared with control (Benbrahim-Tallaa et al. 2007). Thus, there is a correlation between elevated levels of active phosphor-MAPK and arsenic-induced prostate cell transformation.

Prostate cancer is a leading cause of male cancer death because in its advanced stages it acquires androgen independence and becomes resistant to androgen ablation therapy. Surgery can cure locally confined prostate cancer, but there are currently no effective treatments for androgen-independent, metastatic prostate cancer. When prostate cancer progresses in this manner, it is variously called “androgen independent” or “hormone refractory,” because it is resistant to hormone ablation therapy. However, evidence indicates advanced prostate cancers often are not fully independent of androgen, but rather have become hypersensitive even to very low levels of androgen (Weber and Gioeli 2004). A majority of prostate tumors obtained from patients failing androgen ablation therapy overexpress the androgen receptor (AR), sensitizing the cells to low levels of androgen (Linja et al. 2001). This over-expression is often associated with gene amplification (Linja et al. 2001). Frequently, the AR is mutated in advanced prostate cancers, which results in a receptor that can be activated by nonandrogens (Culig et al. 2001). Because the Ras/MAPK signaling pathway can also reduce the androgen requirement of prostate cells (Bakin et al. 2003), one would predict that stimulation of this signaling pathway might allow androgen-regulated gene expression even at very low levels of androgen. Evidence suggests that the Raf/MEK/ERK pathway plays a critical role in the modulation of AR activity in response to *ras* (Gioeli 2005). In addition, MAP kinase activity correlates with progression to an increasingly advanced and hormone-independent stage (Gioeli et al. 1999).

**Figure 1:**
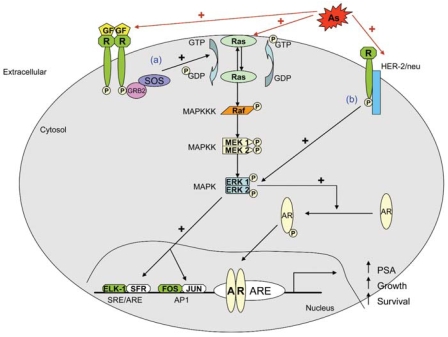
Mechanisms of arsenic-induced acquired androgen independence. Abbreviations: AR, androgen receptor; ARE, androgen responsive element; As, arsenic; GF, growth factors; MAPK, mitogen-activated protein kinase; MAPKK, MAPK kinase; MAPKKK, MAPK kinase kinase; PSA, prostate-specific antigen. It is known that exposure to arsenic initiates GF receptor signaling and Ras-dependent activation of MEK1/2 and ERK1/2. (*A*) As prostate cancer progresses to androgen independence, the growth factors production increases. Growth factor signal transduction pathways have been shown to stimulate AR activation. All these growth factors use the Ras/MAPK pathway for a portion of their signal transduction. Binding of GF results in dimerization, autophosphorylation of the receptor, and tyrosine phosphorylation of other proteins. The GF receptor activates ras which in turn activates Raf, which phosphorylates and activates MEK, which in turn, phophorylates and activates ERK. Activated MAPK can regulate targets in the cytosol and also translocate to the nucleus causing phosphorylation of transcription factors such as c-Fos to create AP-1 and ELK-1, which contribute to proliferation. (*B*) HER-2/neu promotes phosphorylation of AR at multiple sites even in the presence of very low androgen levels. HER-2/neu indirectly activates MAPK. MAPK might phosphorylate the AR, creating an androgen-independent receptor.

CAsE-PE cells, in which chronic arsenic exposure induced malignant transformation, hyperproliferation, and overexpression of K*-ras* (Benbrahim-Tallaa et al. 2005a), have also been used to help define the role of arsenic in prostate cancer progression. The evidence shows CAsE-PE cells clearly acquired androgen independence during transformation that is not associated with AR overexpression (Benbrahim-Tallaa et al. 2005b). The AR in CAsE-PE cells actually is less responsive to androgen, indicating an AR mutation that causes hypersensitivity to androgens is unlikely (Benbrahim-Tallaa et al. 2005b, 2007). In addition, alterations in androgen metabolism, estrogen production, and estrogen receptor levels and sensitivity also had limited roles in this conversion (Benbrahim-Tallaa et al. 2005b, 2007). However, the overexpression of *HER-2/neu* is a prominent feature (Benbrahim-Tallaa et al. 2007) and one in which CAsE-PE cells have in common with androgen-independent human prostate carcinoma (Table 2). Thus, it appears arsenic-induced malignant transformation precipitates upregulation of *ras*, which in turn, allows by-pass of AR to induce androgen independence in human prostate epithelial cells (Figure 1). The fact that a common environmental contaminant such as arsenic can induce prostate tumor cells to progress to a much more lethal state could be very important in human populations exposed to this metalloid.

Overall the CAsE-PE cells and their heterotransplantation tumors show a remarkable series of traits in common with advanced human prostate carcinoma (Table 2).

## Conclusions

It has been known for over a century that inorganic arsenic is a human carcinogen. Arsenic exposure affects millions of people worldwide. Various studies in human populations exposed to arsenic via the environment provide evidence of a causal link to prostate cancer. In many cases this association is dose related, adding further evidence for an etiological role for the metalloid in this important human cancer. Rodent models of inorganic arsenic carcinogenesis generally have been slow to develop and have not specifically shown the prostate as a target of inorganic arsenic carcinogenesis. The rat, which is a species of choice for animal models of human prostate cancer, is unfortunately a poor choice for modeling human arsenic toxicity. Various studies using human prostate epithelial cells in culture have shown that low-level inorganic arsenic exposure can induce malignant transformation specifically in these cells. The finding that human prostate epithelial cells are directly sensitive to malignant transformation induced by inorganic arsenic strongly supports a potential role for arsenic in human prostate cancer. Heterotransplantation of these cells into nude mice produced aggressive carcinomas that overexpress PSA in a fashion similar to human prostate carcinoma. In addition, inorganic arsenic stimulates acquired androgen independence during this malignant transformation, a condition associated with advanced human prostate cancer and poor prognosis. The cancer risk at low doses of arsenic is a subject of considerable debate and may not be solved solely by epidemiologic means, particularly for target sites such as the prostate, for which there are currently no whole-animal models. Therefore, it is essential to learn more about arsenic’s mode of action at the target cell level. Arsenic seems to have the potential for many mechanisms of action in the development of cancer, including prostate cancer. Finally, additional research is clearly needed at all levels on the role of arsenic in prostate cancer development and progression.

## Figures and Tables

**Table 1:** Epidemiologic studies of arsenic exposure and prostate cancer in humans.

Study	Population location	Source of arsenic	Result	Dose–response relationship
Chen et al. 1988	Southwest Taiwan^a^	Drinking water	Increased mortality	Clear evidence
Wu et al. 1989	Southwest Taiwan^b^	Drinking water	Increased mortality	Clear evidence
Chen and Wang 1990	Taiwan	Drinking water	Increased mortality	Clear evidence
Tsai et al. 1999	Southwest Taiwan	Drinking water	Increased mortality	Not investigated
Lewis et al. 1999	Utah, USA	Drinking water	Increased mortality	Some evidence^c^
Hindwood et al. 1999	Victoria, Australia	Local water/soil	Increased incidence	No evidence^d^

a Study focused on the area of endemic blackfoot disease.

b The Wu et al. (1989) study used the Chen et al. (1988) population, with expansion into additional villages in the blackfoot-endemic area.

c Based on the authors’ interpretation after stratification of data based on drinking-water levels.

d The rate of prostate cancer incidence was significantly elevated at the highest level of exposure when arsenic exposure was stratified based on arsenic in water and/or soil. When arsenic exposure was stratified on water levels only (low, medium, high, and very high), prostate cancer incidence appeared elevated in the high and very high categories [see Figure 3 in Hindwood et al. (1999)]. This did not, however, show a significant linear dose–response relationship.

**Table 2:** Characteristics in common between human prostate carcinoma cells and arsenic-transformed human prostate epithelial cells.

Characteristic	Human prostate carcinoma	Arsenic-transformed cells	References
Hyperproliferative	+	+	Achanzar et al. 2001 Benbrahim-Tallaa et al. 2005b
*MMP-9* overexpression	+	+	Achanzar et al. 2001 Benbrahim-Tallaa et al. 2005a
Tumor formation	+	+	Achanzar et al. 2001
*PSA* overexpression	+	+	Achanzar et al. 2001 Benbrahim-Tallaa et al. 2005b Benbrahim-Tallaa et al. 2007
Unmutated *ras* overexpression	+	+	Benbrahim-Tallaa et al. 2005a Benbrahim-Tallaa et al. 2007
Acquired androgen independence through AR by-pass	+	+	Benbrahim-Tallaa et al. 2005b Benbrahim-Tallaa et al. 2007
*HER-2/neu* overexpression	+	+	Benbrahim-Tallaa et al. 2007
Invasive	+	+^a^	Achanzar et al. 2001

a In tumors formed by heterotransplant of CAsE-PE cells.
